# Supplementary material for: Drug Resistance Mechanism of M46I-Mutation-Induced Saquinavir Resistance in HIV-1 Protease Using Molecular Dynamics Simulation and Binding Energy Calculation
Source: Viruses. 2022 Mar 28;14(4):697. doi: 10.3390/v14040697 (PMC9031992; doi:10.3390/v14040697)
Supplement: Supplementary file 1 [file viruses-14-00697-s001.zip › viruses-1610853-supplementary.pdf]

Supplementary Table S1: Distance between amino acid residues at specific time interval of the 100 ns molecular dynamics simulation period (individual and averaged). Additionally average value during the entire period is also tabulated.

**Time (ns)**	**Asp25-Asp124 (nm)**	**Asp25-Ile50 (nm)**	**Asp25-Ile149 (nm)**
**Unbound wild type protease (WT)**			
1	0.69	1.35	1.16
20	0.70	1.38	1.25
40	0.67	1.36	1.15
60	0.68	1.82	1.33
80	0.65	13.6	1.42
100	0.64	1.12	1.32
**Average**	0.67	1.39	1.27
**1-100 ns MD Average Distances**	0.6529±0.02235	1.4359±0.1914	1.3359±0.1837
**Unbound M46I mutation carrying protease (MI)**			
1	0.7	1.47	1.29
20	0.64	1.64	2.15
40	0.67	1.73	2.23
60	0.72	1.79	2.16
80	0.71	1.58	2.42
100	0.65	1.61	2.18
**Average**	0.68	1.63	2.071
**1-100 ns MD Average Distances**	0.6838±0.0338	1.7026±0.2291	2.0923±0.2797
**Saquinavir bound wild type protease (SQ-WT)**			
1	0.73	1.50	1.28
20	0.68	1.24	1.48
40	0.65	1.52	1.63
60	0.71	1.45	1.93
80	0.66	1.41	1.82
100	0.67	1.58	1.70
**Average**	0.68	1.45	1.64
**1-100 ns MD Average Distances**	0.6659±0.0215	1.5600±0.1306	1.7312±0.1398
**Saquinavir bound M46I protease (SQ-MI)**			
1	0.72	1.45	1.28
20	0.70	1.79	2.21
40	0.80	2.16	2.01
60	0.90	1.71	2.18
80	0.87	1.71	2.20
100	0.76	2.29	2.20
**Average**	0.79	1.85	2.013
**1-100 ns MD Average Distances**	0.7843±0.0715	1.8856±0.2939	2.0348±0.3014

Supplementary Figure S1

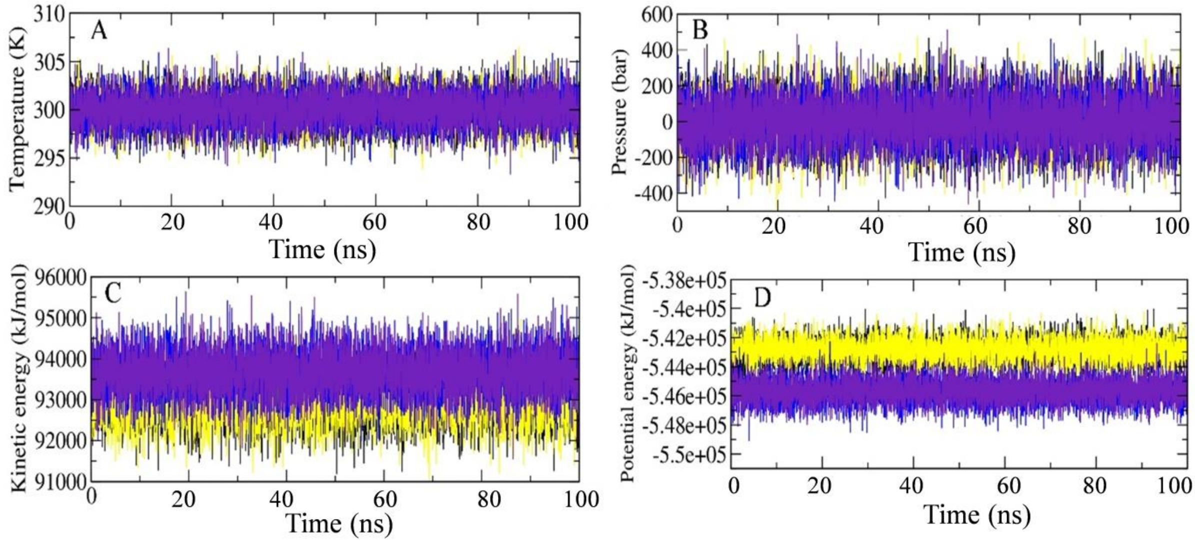

Supplementary Figure S1: Temperature (**A**), pressure (**B**), Kinetic energy (**C**), and potential energy (**D**) of WT (Black), MI (Yellow), SQ-WT (Blue), and SQ-MI (Purple) simulation systems. WT- Wild type, SQ-WT- Saquinavir bound wild type, MI- M46I mutation carrying protease, and SQ-MI- Saquinavir bound mutation carrying protease.

Supplementary Figure S2

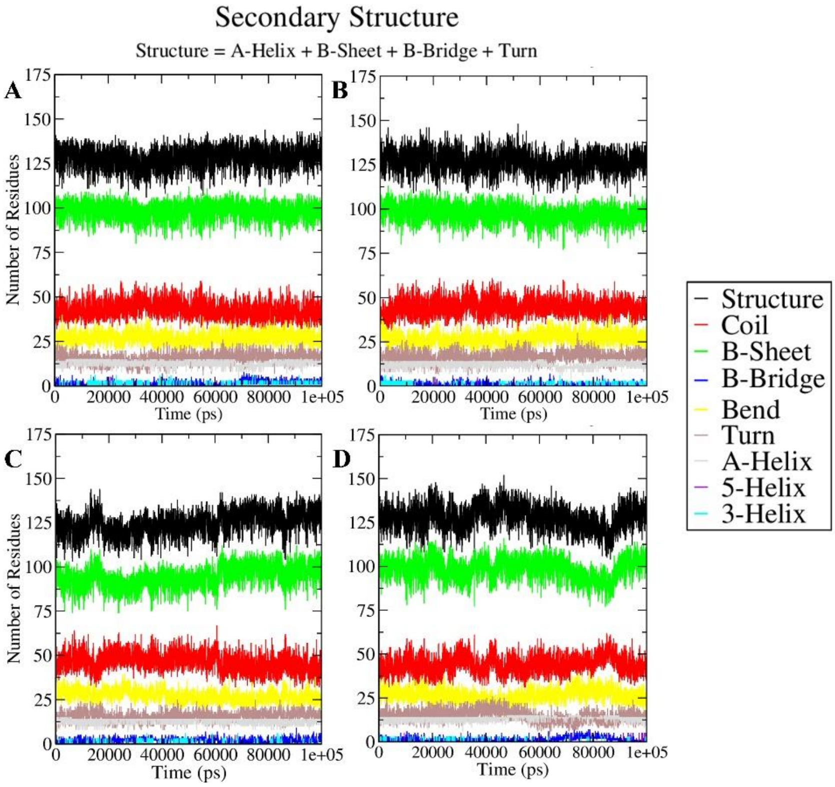

Supplementary Figure S2: Evolution of secondary structure during 100 ns MD simulation for WT (**A**), MI (**B**), SQ-WT (**C**), and SQ-MI (**D**). WT- Wild type, SQ-WT- Saquinavir bound wild type, MI- M46I mutation carrying protease, and SQ-MI- Saquinavir bound mutation carrying protease.

Supplementary Figure S3

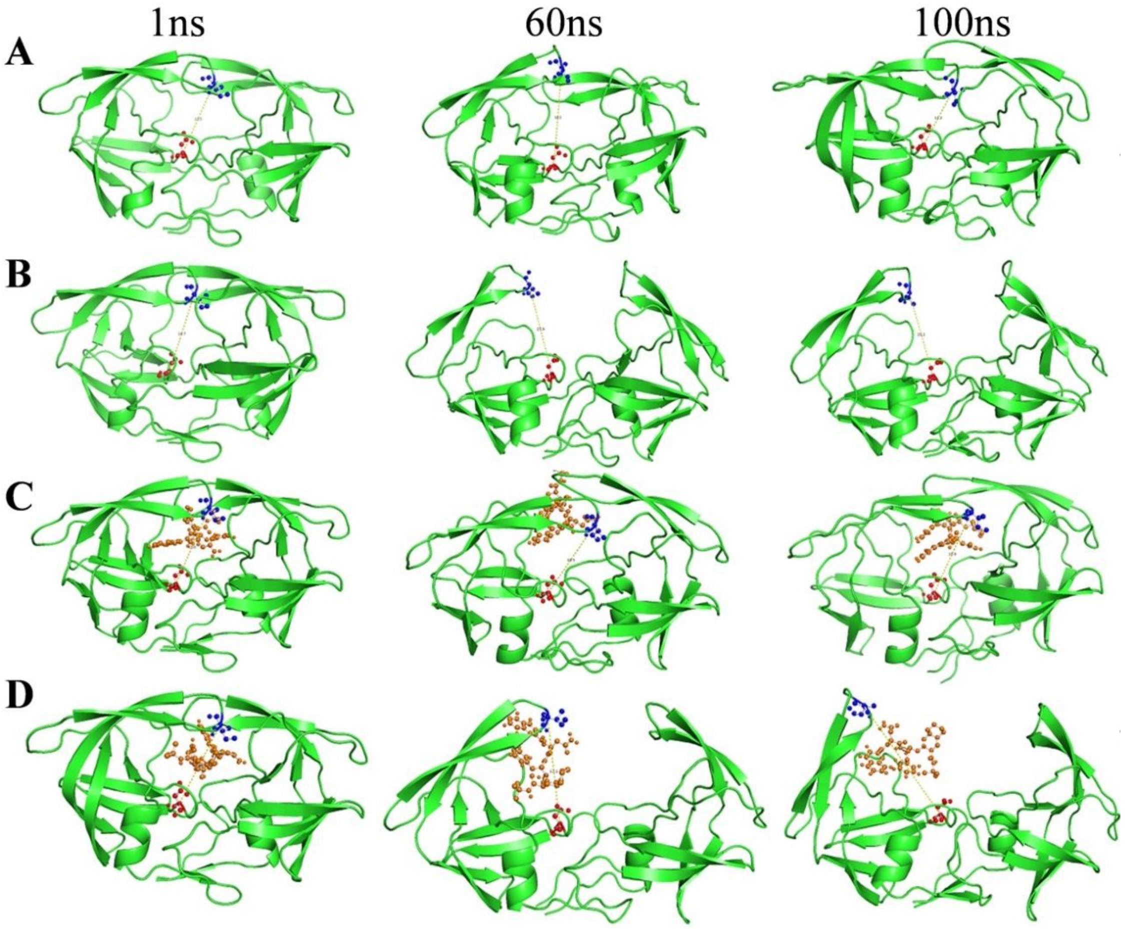

Supplementary Figure S3: Distance between Asp25-Ile50 amino acid residues in (**A**) WT, (**B**) MI (**C**) SQ-WT and (**D**) SQ-MI systems at 1, 60 and 100 ns simulation period. WT- Wild type, SQ-WT- Saquinavir bound wild type, MI- M46I mutation carrying protease, and SQ-MI- Saquinavir bound mutation carrying protease.

Supplementary Figure S4

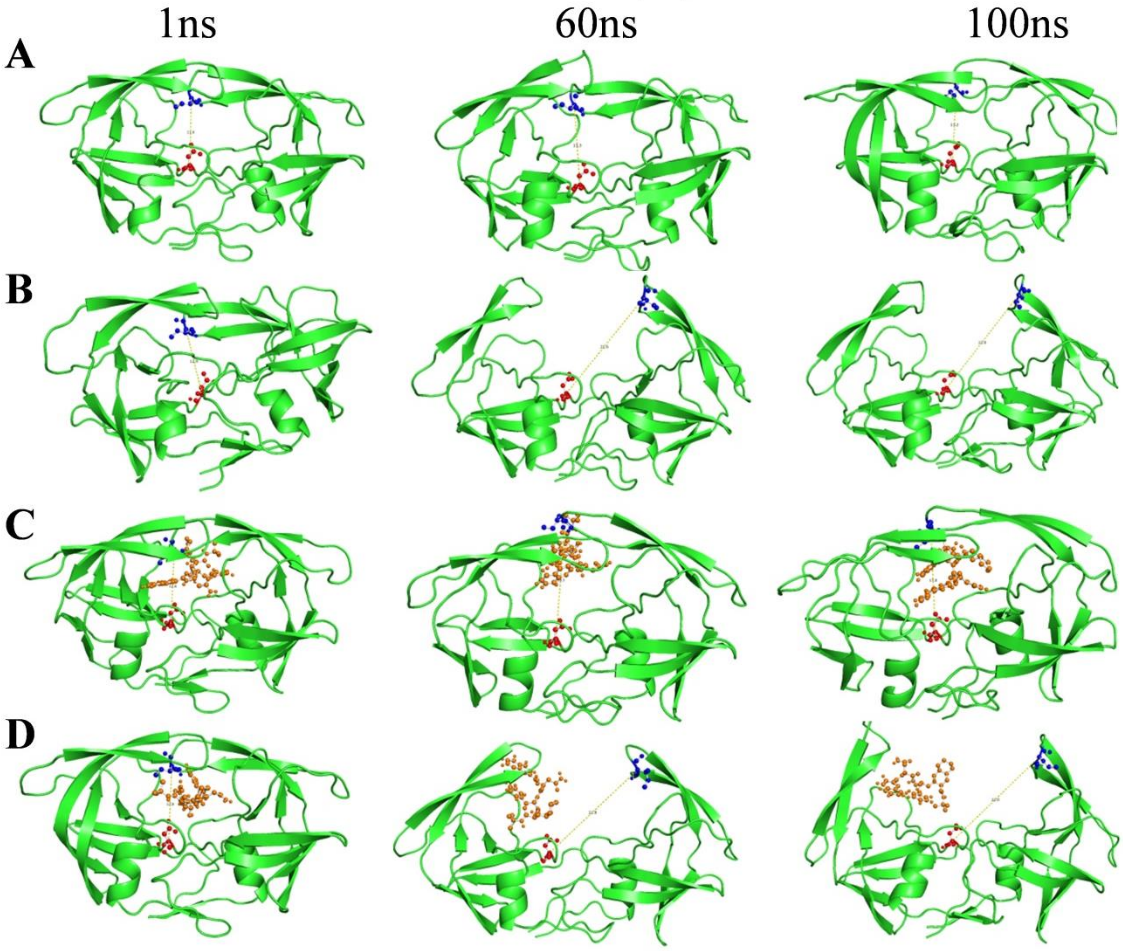

Supplementary Figure S4: Distance between Asp25-Ile149 amino acid residues in (**A**) WT, (**B**) MI (**C**) SQ-WT and (**D**) SQ-MI systems at 1, 60 and 100 ns simulation period. WT- Wild type, SQ-WT- Saquinavir bound wild type, MI- M46I mutation carrying protease, and SQ-MI- Saquinavir bound mutation carrying protease.

Supplementary Figure S5

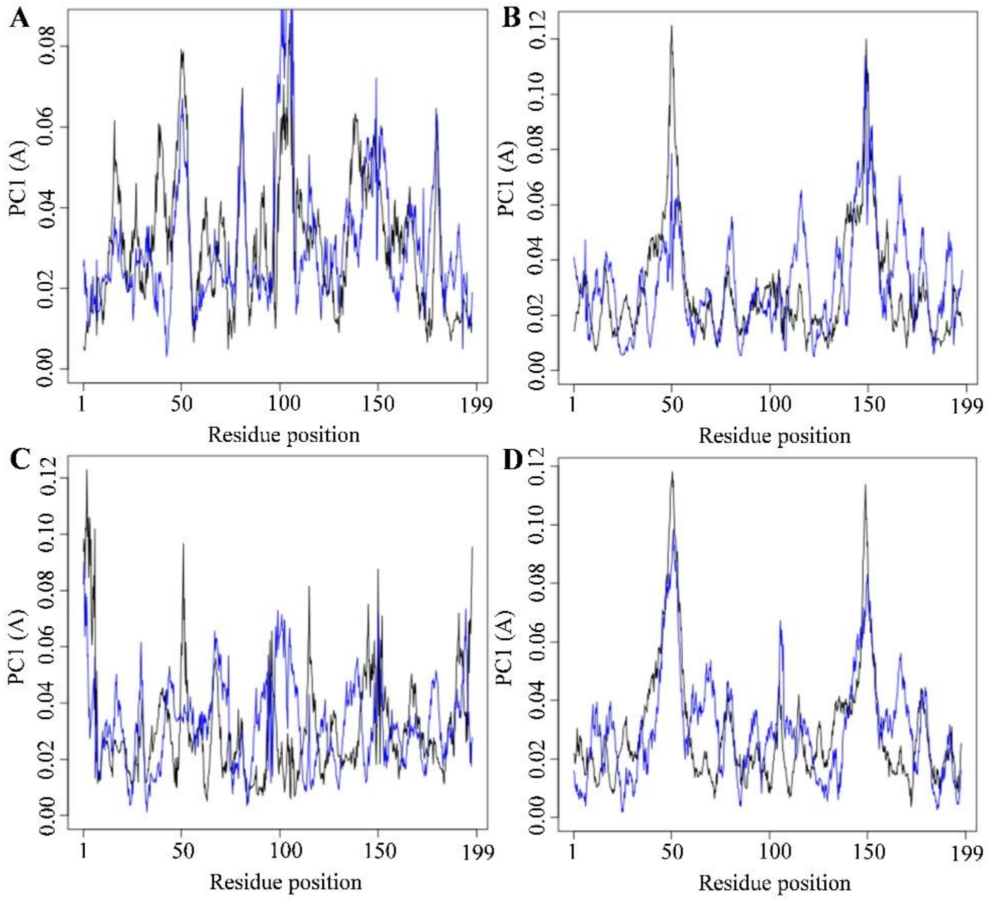

Supplementary Figure S5: Projection of PC1 (Blue) on RMSF (Black) during 100 ns MD simulation for WT (**A**), MI (**B**), SQ-WT (**C**), and SQ-MI (**D**). WT- Wild type, SQ-WT- Saquinavir bound wild type, MI- M46I mutation carrying protease, and SQ-MI- Saquinavir bound mutation carrying protease.

Supplementary Figure S6

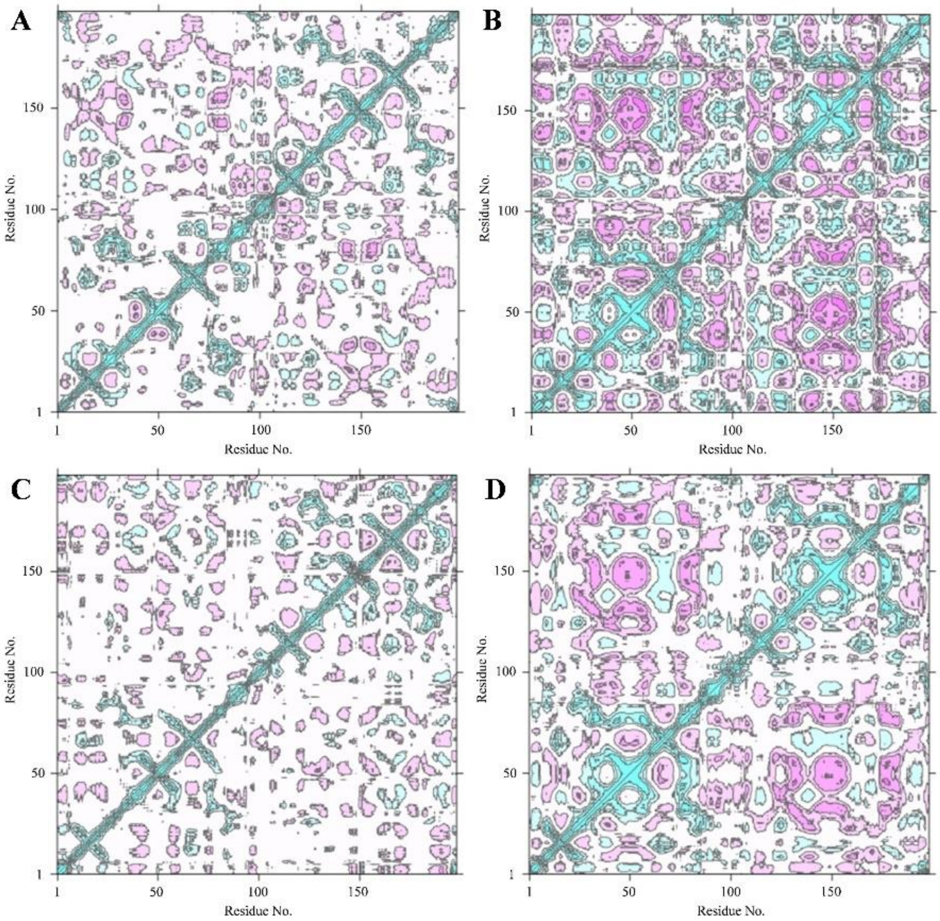

Supplementary Figure S6: Dynamic cross correlation matrix (DCCM) of test systems during 100 ns MD simulation for WT (**A**), MI (**B**), SQ-WT (**C**), and SQ-MI (**D**). WT- Wild type, SQ-WT- Saquinavir bound wild type, MI- M46I mutation carrying protease, and SQ-MI- Saquinavir bound mutation carrying protease.
